# Heat Strain Evaluation of Power Grid Outdoor Workers Based on a Human Bioheat Model

**DOI:** 10.3390/ijerph19137843

**Published:** 2022-06-26

**Authors:** Letian Li, Boyang Sun, Zhuqiang Hu, Jun Zhang, Song Gao, Haifeng Bian, Jiansong Wu

**Affiliations:** 1 School of Emergency Management and Safety Engineering, China University of Mining and Technology-Beijing, Beijing 100083, China; sqt2010104042@student.cumtb.edu.cn (L.L.); sqt2010104045@student.cumtb.edu.cn (B.S.); sqt1910101019@student.cumtb.edu.cn (Z.H.); 2 Grid Development Integrated Research Department, State Grid Energy Research Institute Co., Ltd., Beijing 102209, China; zhangjun@sgeri.sgcc.com.cn (J.Z.); bianhaifeng@sgeri.sgcc.com.cn (H.B.); 3 Safety Supervision and Quality Department, State Grid Liaoning Electric Power Supply Co., Ltd., Shenyang 110006, China; 13889286262@163.com

**Keywords:** occupational health and safety, grid workers, heat strain, thermophysiological responses, acceptable maximum working time

## Abstract

Power grid outdoor workers are usually exposed to hot environments and could suffer the threats to occupational health and safety like heat strain and injury. In order to predict and assess the thermophysiological responses of grid workers in the heat, the clothing thermal insulation of grid worker ensembles was measured by a thermal manikin and a multi-segment human bioheat model was employed to evaluate the thermophysiological response parameters of grid workers such as core temperature, skin temperature and sweat loss. The results show that working in a hot environment can cause a obvious increase in core temperature and skin temperature of grid workers, and the acceptable maximum working time of grid workers varies greatly in different hot environments. A reasonable work organization strategy can effectively decrease the core temperature and sweat loss, increasing the duration of acceptable maximum working time for grid workers. This study is helpful to assess heat-related risks of grid workers and support power grid companies to rationalize work organization strategies and personal protection guidelines.

## 1. Introduction

Global climate change has exacerbated the occurrence of extreme heat events around the world, and the emerging trends of increased frequency, intensity and duration of heat waves are being reported [1,2,3]. These changes expose outdoor workers to more heat exposure and increase their risk of heat stress. It is reported that approximately 30% of the world’s population is exposed to heat-related health risks at least 20 days a year, reaching 50% by the end of this century [4]. Heat stress is a well-known occupational health hazard and is prevalent in many industries [5], which has become a serious challenge to occupational health and safety. The combination of external heat and internal heat production from muscular physical activity not only reduces workers’ cognitive performance and capacity for work, but also causes heat stroke, dehydration, heat exhaustion, and other heat-related diseases [6,7]. More importantly, exposure to extreme heat takes broader socio-economic impacts, such as labor productivity losses, inadequate social well-being and higher economic burdens [8,9]. Heat stress faced by production workers has received widespread attention [7,10,11,12], especially in the construction and agricultural industries [13,14], where large numbers of outdoor workers are directly exposed to high temperatures. In the power industry, electricity networks maintain the functioning and stability of society through the transmission of power energy. Field operation tasks of the power grid mainly involve equipment inspection, equipment maintenance, switching operation, emergency repair, etc. Working at heights like this outdoors is often exposed to thermal radiation. Nowadays, a large amount of inspection and maintenance work is still performed by grid outdoor workers. The continuous expansion of the construction scale of electricity networks has brought severe challenges to grid workers. Even though grid workers are usually exposed to high-temperature environments for a long time during the summer, resulting in heat-related diseases, there have been few researchers focusing on the heat strain experienced by the electrical utility workers, but the fact is that the power industry faces occupational heat stress that threatens the safety and health of workers like the environment of other outdoor labor industries [15,16,17]. Therefore, it is of great significance to assess the thermophysiological response of grid workers in high temperature environments in time and to give early and proactive warning to those who may be at risk of heat stress.

The evaluation of the physiological strain of electrical utility workers is still in the field monitoring stage. Brearley et al. [15] and Meade et al. [16] assessed the thermophysiological responses experienced by Australian and North American grid workers performing operations in hot environments by monitoring the physiological indicators such as core temperature, heart rate, urine specific gravity and sweat rate, respectively. The results showed that electric utility workers experienced severe environmental heat stress including excessive core temperature and dehydration. Combined with the physiological strain assessment of grid workers during their shifts [18], the results of Meade et al. showed that performing tasks with high labor levels or continuous shift work can exacerbate the heat stress of grid workers. Moreover, electrical utility workers who participated in the test may not carry out self-pacing as a strategy to ease their heat stress [16]. The results of this series of field monitoring and experimental studies have provided valuable data for the evaluation and management of heat stress in grid workers, but it is still not enough. On-site monitoring does not cover the entire workers, and the human thermophysiological response is influenced by several important factors, including age, gender, and body mass index (BMI) [19,20,21,22]. Therefore, even when performing the same task, different grid workers are subject to different levels of heat stress. In addition, the above study involved a limited number of field operating environments. In extreme environments with high temperatures and humidity, the heat stress risk experienced by grid workers increases because the heat dissipation ability is affected [23]. Therefore, accurate assessment of the physiological strain and heat stress of grid workers requires comprehensive methods.

In order to evaluate the impact of hot environments on the human body, many scholars have formulated or revised a large number of heat stress and heat strain indices in the past decades. For example, the wet-bulb globe temperature index (WBGT) widely used as an ISO standard [24], extended SET proposed by Wonseok [25], Environmental Stress Index (ESI) proposed by Moran [26], Outdoor Environmental Heat Index (OEHI) proposed by Golbabaei [27]. However, these heat stress assessment indexes are calculated based on environmental parameters such as air temperature, humidity, and thermal radiation, and they only consider the effects of the environment on human heat stress and do not reflect the responses and thermoregulation of the human body to the environment. In fact, such as local heat sources, metabolic heat production within the human body, all affect the exchange of heat between the body and the environment. Besides, various urban microclimate models developed based on CFD, such as Envimet, PALM and MITRAS, are also effective tools for assessing huamn heat stress [28,29,30,31]. Since such models well simulate the heat exchange between the human body and the environment, if combined with advanced human bioheat models, assessment of core temperature elevation and water loss considering the realistic ambient environment can be achieved [32]. A reliable occupational heat stress index should consider not only workplace environmental conditions but also activity levels and clothing properties [10]. The thermoregulation model can accurately simulate human thermophysiological responses by introducing the heat generation and heat exchange mechanisms of metabolism, respiration, skin blood flow and sweating, and considering the heat loss related to clothing and environment, it is widely used in predicting human thermophysiological responses therefore [33,34,35,36]. For example, the Predicted Heat Strain (PHS) model proposed by Malchaire et al. [37] has been used to evaluate occupational heat stress [38,39] as an ISO standard [40]. However, the predictive accuracy of the model on high insulation clothing has been criticized [41]. By contrast, the advanced multi-segment human thermoregulation model can reasonably predict the human body’s core temperature, skin temperature, sweat rate and other thermophysiological parameters in a hig-temperature environment, which can more accurately evaluate the heat stress level of outdoor workers [42].

Outdoor workers in different industries are exposed to different heat strain, sothe evaluation of occupational heat stress should be based on an appropriate method according to the characteristics of different occupations and realistic environments. This study measured the clothing thermal insulation of power grid summer ensembles and a multi-segment human bioheat model was applied to calculate the physiological responses of grid workers such as core temperature, skin temperature and sweat loss. Considering the special characteristics of the grid outdoor work, we have selected the appropriate metabolic rate and environmental scenarios. The objective of this study is to evaluate the heat stress level of grid workers and optimize the work organization strategies based on acceptable maximum working time (AMWT) in different hot environments. The results could be a certain reference for preventing heat-related injuries, promoting occupational safety, sustaining operations, and maximizing the physical performance of grid workers.

## 2. Methodology

### 2.1. Human Bioheat Model

In this paper, a multi-segment human bioheat model proposed by Tanabe et al. [43] was used to simulate the thermophysiological responses of grid workers under different hot environments. Based on the previous studies [44,45], the model named JOS-3 newly introduced the effects of brown adipose tissue activity, age and solar radiation (including long-wave radiation and short-wave radiation). Moreover, although the effect of *Q*_10_ effect was not considered [46], calculations of sweating and basal metabolic heat production were improved and individual characteristics such as height, weight and age on the body’s thermophysiological response were also taken into account. The model has been validated in a variety of steady and transient thermal environments and has been used by Tang et al. [47] to analyze human skin temperature and thermal sensation in conjunction with measured thermal insulation of clothing.

The multi-segment human bioheat model consists of two parts: passive system and active system. In the passive system of the JOS-3 model, the human body is divided into 17 segments corresponding to the head, neck, back, chest, pelvis, shoulders, arms, hands, thighs, legs, and feet. Different segments are divided into different layers since the different distribution of internal organs and bones in the human body. The body segments of the head and pelvis both include six layers: skin, fat, muscle, core, artery and vein. The neck, back and chest have four layers: skin, core, artery and vein. The segments of shoulders, arms, hands, thighs, legs and feet include five layers: skin, core, artery, skin, and superficial vein. Since each layer of each segment can be regarded as a node, the human bioheat model has a total of 85 nodes including a central blood compartment, which is set up to exchange heat between each node and itself. The passive system includes not only the division of the human body mentioned above but also the heat transfer process inside the human body and the heat exchange process between the human body and the ambient environment by convection, radiation and evaporation. The active system is expressed as the body’s processes of thermoregulation, including vasodilation, vasoconstriction, sweating and shivering. These modes of thermoregulation are controlled by the hypothalamus and are associated with error signals. The error signals are expressed as the difference between the temperature of each node and the set point temperature, and it is calculated as shown below.

(1) $$Err_{j,i}=T_{j,i}-Tsetpt_{j,i}$$

where *Err_i,j_* is the error signals of node (*i*, *j*), °C; *T_i,j_* and *Tsetpt_i,j_* are the temperature of setpoint temperature of node (*i*, *j*), respectively, °C. The detailed information of the thermoregulation process equations can be acquired in [43].The body temperature will maintain stability when there is a heat balance between the human body and its external environment [23], that is, the heat generated and stored by the human body is equal to the heat lost to the external environment. The heat balance equations are used to describe the dynamic heat exchange process. In the human bioheat model used in this paper, the heat balance equations of skin, core, artery, vein and superficial vein are expressed in Equations (1)–(5), respectively.

(2) $$c_{ar,i}\frac{dT_{ar,i}}{dt}=B_{ar,i}-D_{ar-cr,i}-D_{ar-ve,i}$$

(3) $$c_{ve,i}\frac{dT_{ve,i}}{dt}=B_{ve,i}-D_{ve-cr,i}-D_{ar-ve,i}$$

(4) $$c_{sve,i}\frac{dT_{sve,i}}{dt}=B_{sve,i}-D_{sve-sk,i}$$

(5) $$c_{cr,i}\frac{dT_{cr,i}}{dt}=\{\begin{array}{ll} Q_{cr,i}+B_{cr,i}-D_{cr-ms,i} & \;(\mathrm{Head}\;\mathrm{and}\;\mathrm{Hip})\;\\ Q_{cr,2}+B_{cr,2}-D_{cr-sk,2}-RES & \;(\mathrm{Chest})\;\\ Q_{cr,i}+B_{cr,i}-D_{cr-sk,i} & \;(\mathrm{Neck}\;\mathrm{and}\;\mathrm{Back})\;\\ Q_{cr,i}+B_{cr,i}+D_{ar-cr,i}+D_{ve-cr,i}-D_{cr-sk,i} & \;(\mathrm{Limb})\; \end{array}$$

(6) $$c_{sk,i}\frac{dT_{sk,i}}{dt}=\{\begin{array}{ll} Q_{sk,i}+B_{sk,i}+D_{fat-sk,i}-\left(C_{0}+R_{0}\right)-E_{0}+SW_{sk,0} & (\mathrm{Head}\;\mathrm{and}\;\mathrm{Hip})\;\\ Q_{sk,i}+B_{sk,i}+D_{cr-sk,i}-\left(C_{i}+R_{i}\right)-E_{i}+SW_{sk,i} & (\mathrm{Neck},\;\mathrm{Chest}\;\mathrm{and}\;\mathrm{Back})\;\\ Q_{sk,i}+B_{sk,i}+D_{cr-sk,i}+D_{sve-sk,i}-\left(C_{i}+R_{i}\right)-E_{i}+SW_{sk,i} & (\mathrm{Limb})\; \end{array}$$

where *c_i,j_* is the heat capacity of node (*i*, *j*), J/K; *T_i,j_* is the temperature, °C; *t* is the time, s; *Q_i,j_* is the heat production, W; *B_i,j_* is the heat exchange caused by blood flow, W; *D_i,j_* is the heat exchange by conduction, W; *RES* is the heat exchange by respiration only at chest, W; *C_i,j_* and *R_i,j_* are the heat exchange by convection and radiation between the skin and the external environment, respectively, W; *E_i,j_* is the evaporative heat exchange, W; *SW_i,j_* is the heat gain by shortwave solar radiation at the skin, W.

The heat balance equation of the central blood compartment is given by

(7) $$c_{cb}\frac{dT_{cb}}{dt}=B_{cb}$$

where *c_cb_* and *T_cb_* are the heat capacity and the temperature of the central blood compartment, respectively.

The model has been coded using Python and its package is open-sourced. With the input of four environmental parameters: ambient temperature, mean radiation temperature, relative humidity, and wind speed, as well as four individual parameters: height, weight, age, and physical activity ratio, and the local thermal insulation of the covering clothes, the changes in physiological parameters such as core temperature and skin temperature of each part of the human body can be calculated for a specific scenario. These physiological parameters will be used to evaluate the level of heat strain that the power grid outdoor workers are exposed to in a given scenario.

The outdoor operation activities of the power grid are diverse and complex, often including power line inspection and maintenance, transmission tower maintenance, transmission line erection, substation construction, etc. When power grid workers are engaged in different tasks, the metabolic rate will also vary, resulting in different thermophysiological responses. However, few literatures have reported the exact values of these metabolic rates. Ainsworth et al. reported a human metabolic equivalent of 3.3 met when hooking up wires [48]. Based on this, the metabolic rate was set to 3.3 met when using the JOS-3 model to evaluate the thermophysiological responses of grid workers. As one of the important parameters, the clothing thermal insulation measured by thermal manikin was used as the input parameter of the model. The individualized parameters use the default values of the model, i.e., a height of 1.70 m and a weight of 60 kg, which is also similar to the average body size of Chinese grid workers. The environmental scenario was determined by referring to the historical statistics provided by State Grid Electric Power Supply Co., Ltd. (Beijing, China) and previous studies [15,16], and we found that the ambient temperature when heat stroke occurred in grid outdoor workers was above 30 °C. Therefore, the temperature of the thermal environment to be simulated in this paper was all set above 30 °C. In this paper, OriginPro 2021b (Northampton, USA) was used to process and plot the calculated data.

### 2.2. Measurement of Power Grid Ensembles Insulation

#### 2.2.1. Thermal Manikin and Power Grid Ensembles

The thermal manikin Newton (Measurement Technology Northwest, Seattle, USA) was used for measuring clothing thermal insulation of power grid ensembles following the methods described in standard ISO 15831 [49]. The thermal manikin has a similar size to an average Asian adult male (height: 168.5 cm) and can be operated in a temperature range of −20 °C to 45 °C with a measurement accuracy of ±0.1 °C. The temperature and heat flux of each zone could be set up and recorded independently with the supporting software ThermDAC (Measurement Technology Northwest, Seattle, USA) [50]. In this study, the skin surface temperature of the thermal manikin was kept constant at 34 °C according to standard ISO 15831 during measurement and the thermal insulation was calculated by recording the heat flux through each body part [49].

**Figure 1:**
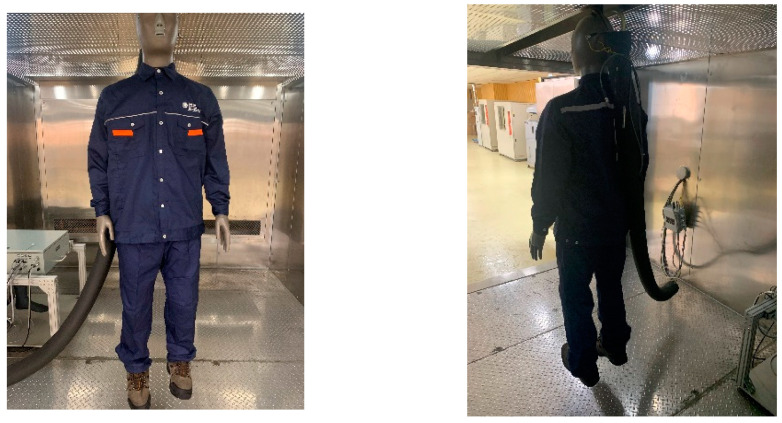
The thermal manikin Newton with power grid ensembles.

The measured clothing on the thermal manikin was summer ensembles for power grid outdoor workers acquired from State Grid Corporation of China (Beijing, China), consisting of a long-sleeve coat, a pair of trousers, and a pair of protective shoes, as shown in Figure 1. Most of the coats and trousers were made of cotton and the shoe soles were made of rubber. During the test, the thermal manikin remained standing and stationary while wearing the power grid ensembles.

#### 2.2.2. Test Procedure

The clothing thermal insulation measurement was carried out in a climate chamber (6 m × 5 m ×2.7 m) at the China National Institute of Standardization. The temperature and humidity of the climate chamber can be controlled independently. The environmental temperature can be adjusted in the range of 18~48 °C and with an accuracy of ±0.5 °C. The environmental humidity can be adjusted in the range of 30%~80% with an accuracy of ±5%. The air conditioning system ensures uniform and stable air temperature, humidity and wind speed in the chamber. During the test, the ambient temperature of the climate chamber was set to 24 °C, the relative humidity was set to 47%. The wind speed was kept at 0.4 m/s according to ISO 15831 [49]. After the skin temperature of the thermal manikin had stabilized, the thermal insulation of the naked manikin and clothed manikin with power grid ensembles was measured, respectively. The thermal insulation of the head was not measured in this experiment as the control circuit entered through the head of the thermal manikin and it was not convenient to put a helmet on the manikin. The test was carried out twice and each lasting 30 min, the heat flux and temperature of each body part were recorded at one-minute intervals, and the average of the two tests was taken as the final result.

#### 2.2.3. Calculation of Thermal Insulation

The thermal insulation was calculated according to the serial model method described in ISO 15831 [49]. The total thermal insulation and air insulation were calculated from the local thermal insulation of different body parts, and then the basic thermal insulation of power grid ensembles was obtained. The calculation of thermal insulation is given by:

(8) $$I_{t}=\sum_{i}f_{i}\times \left[\frac{\left(T_{si}-T_{a}\right)\times a_{i}}{H_{Ci}}\right]$$

(9) $$I_{a}=\sum_{i}f_{i}\times \left[\frac{\left(T_{si}-T_{a}\right)\times a_{i}}{H_{ci}}\right]$$

(10) $$f_{i}=\frac{a_{i}}{A}$$

(11) $$I_{\mathrm{cl}}=I_{t}-\frac{I_{a}}{f_{\mathrm{cl}}}$$

where *I*_t_ and *I*_a_ are the total thermal insulation of the clothing ensemble and the boundary air layer, respectively, m^2^ K/W. *I*_cl_ is the basic thermal insulation of the clothing ensemble, m^2^ K/W. *T*_s*i*_ and *T*_a_ are the local surface temperature of section *i* of the manikin and the air temperature, K. *a_i_* and *A* are the surface area of section *i* and the whole body of the manikin, m^2^. *H*_c*i*_ is the local heat loss from section *i* of the manikin, W. *f_i_* is the area factor of section *i* of the nude manikin and *f*_cl_ is the clothing area factor.

## 3. Results and Discussion

### 3.1. Thermal Insulation of Power Grid Ensembles

**Figure 2:**
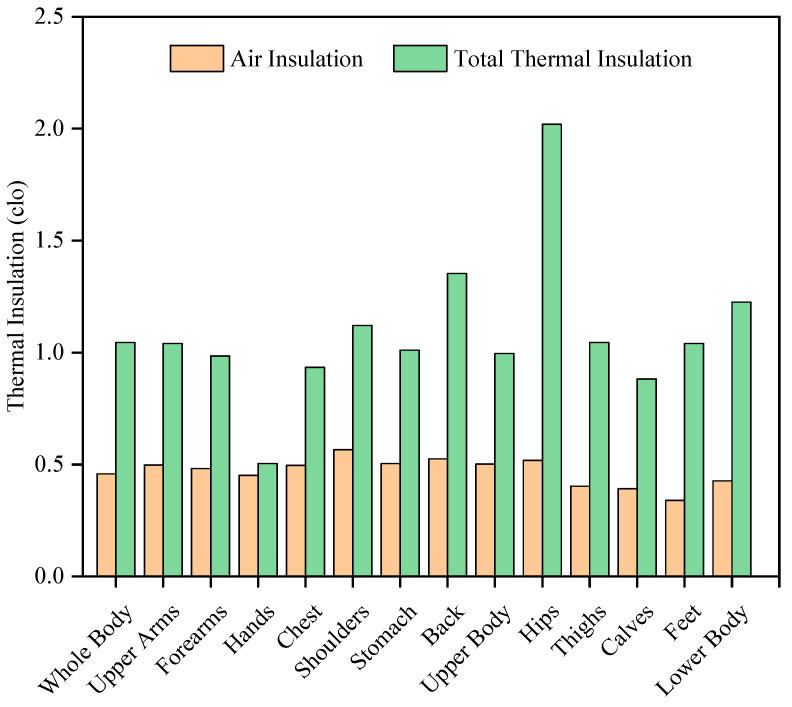
Total thermal insulation of power grid ensembles and air insulation at different body parts.

The total thermal insulation of power grid ensembles and air insulation at different body parts are shown in Figure 2. To calculate the basic thermal insulation, the clothing area factor was determined as 1.36 based on ISO 9920 [51] regarding garments with similar clothing composition. The total thermal insulation and air insulation for the whole body are 1.046 clo and 0.458 clo respectively. Accordingly, the basic thermal insulation of the tested power grid ensembles is 0.709 clo.

The local thermal insulation of different body segments is different. Since the hands were not clothed, the total thermal insulation of these parts is close to the air insulation. Moreover, the total thermal insulation of the shoulders, stomach, back, hips and thighs are higher, and it is almost twice the whole body’s total thermal insulation in the hips. This is because the coat and trousers overlap at the hips. In contrast, the air gap in the chest and calves is larger, so the total thermal insulation of these body parts is also relatively low compared to the whole body total thermal insulation. The total thermal insulation of other body parts is closer to that of the whole body, with a difference of ±10% or less. It is worth noting that although the material of the upper and lower body of the power grid ensembles is the same, the ratios of the total thermal insulation to air insulation of the lower body, such as the hips and thighs, are greater than those of the whole body, making the total thermal insulation of the lower body higher than that of the upper body, which may cause thermal discomfort in these body parts for grid workers in a hot environment. The total thermal insulation of the feet rises to more than three times when naked, which may exacerbate the rise in temperature and increase in sweating of the feet when working in high temperatures, causing discomfort to the power grid workers.

### 3.2. Evaluation of Thermophysiological Responses for Grid Workers

#### 3.2.1. Core Temperature

**Figure 3:**
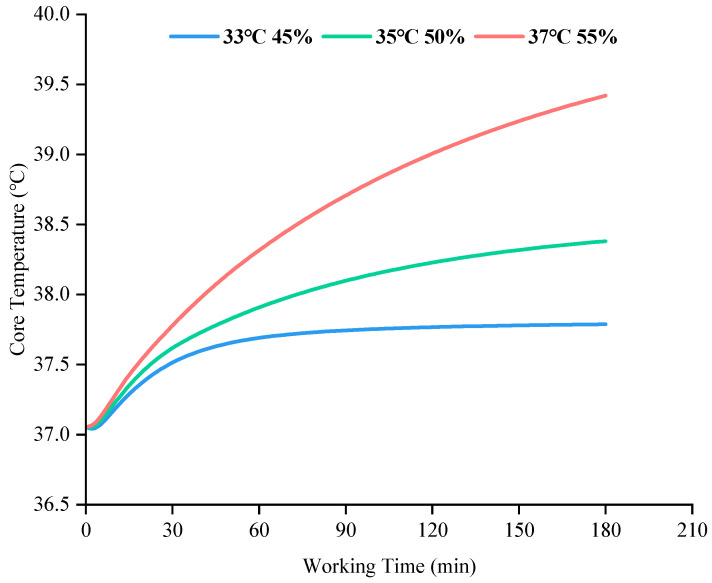
The predicted core temperature of grid workers in different hot environments.

Core Temperature is an important indicator for assessing human heat stress. Figure 3 shows the changes in core temperature of grid workers working in three different high temperature environments for 180 min. At relatively low temperature and humidity (Ta = 33 °C, RH = 45%), the grid workers reached a thermal equilibrium state at approximately 70 min and thereafter the core temperature stabilised at around 37.7 °C. In such an environment, grid workers can work continuously for a long time without exposing themselves to the heat stress risk if they keep themselves well hydrated and reasonably rested. With the further increase in ambient temperature and relative humidity (Ta = 35 °C, RH = 50%), the core temperature of the workers also rose, eventually reaching a new steady state at a higher level. However, in a relatively high temperature and humidity environment (Ta = 37 °C, RH = 55%), the core temperature of grid workers rose rapidly, by 1 °C within 40 min, after which the rate of increase slowed down due to the human thermoregulation mechanism, but still failed to reach a thermal equilibrium state. For this reason, it is important to avoid grid outdoor workers hooking up wires under the environmental condition.

#### 3.2.2. Skin Temperature

Clothing is an intermediate medium between the skin and air, influencing convective, radiant, and evaporative heat exchange between the human body and the ambient environment. The skin temperature can well reflect the thermal state of the human body, and it is an important index to evaluate human thermal sensation and thermal comfort. The large changes in skin temperature are affected by environmental transient changes [52,53]. The changes in human skin temperature can reflect the effect of clothing on human thermophysiological responses.

**Figure 4:**
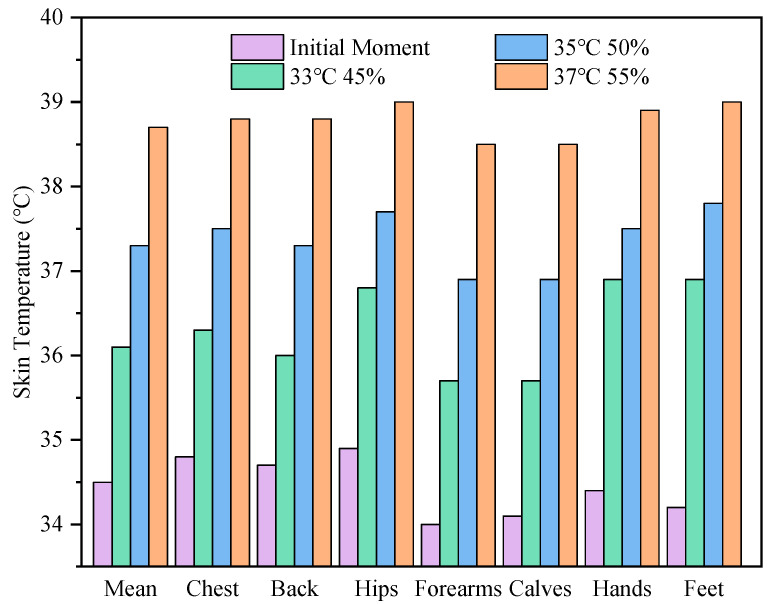
The predicted mean and local skin temperatures of grid workers at the initial and final moments of work in different hot environments.

**Figure 5:**
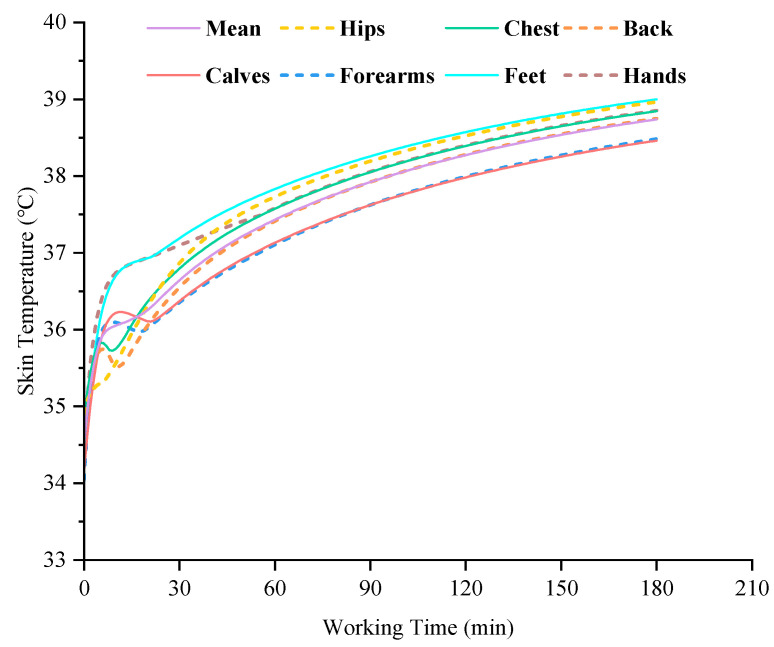
Changes in predicted skin temperatures of grid workers when working at a 37 °C and 55% RH environment.

The mean skin temperature and local skin temperatures of grid workers after working in three different high-temperature environments for 180 min are shown in Figure 4. It can be seen that after working in all three different high-temperature environments for 180 min, the mean skin temperature and local skin temperatures of grid workers were obviously higher than the skin temperatures at the initial moment. The mean skin temperature increased by 1.6 °C, 2.8 °C and 4.2 °C in the three environments, respectively. In particular, the local skin temperatures of all seven body parts also increased by more than 4 °C at 37 °C and 55% relative humidity. As for the local skin temperature, the skin temperature is highest on the hips, almost the same on the forearms and calves, closer on the hands and feet, and slightly higher on the chest than on the back. In addition, the skin temperature of the extremity areas was lower than that of the torso at the initial moment, while after 180 min of work, the skin temperature of the hands and feet increased obviously and was close to that of the hips. Moreover, with the increase in ambient temperature and relative humidity, the local skin temperature of these body parts was closer to the mean skin temperature. The maximum difference between the local skin temperatures and the mean skin temperature in the three high-temperature environments were 0.8 °C, 0.5 °C, and 0.3 °C, respectively. Figure 5 shows the variation in skin temperatures over time for grid workers working in an environment of 37 °C and 55% relative humidity. Unlike the reaching a thermal equilibrium state, the mean skin temperature and the local skin temperatures still had a rising trend at 180 min, indicating that the heat dissipated to the external environment by the workers in this environment was not enough to offset the heat generated by the body, resulting in a generalized temperature increase.

Combining the measured local thermal insulation of the power grid ensembles and the local skin temperatures of the grid workers in high-temperature environments, some suggestions can be made for the design and improvement of power grid ensembles. Parts with high thermal insulation or a high local skin temperature are the focus of attention, such as the chest, back, hips, and feet. In addition to the common methods of using breathable fabrics to increase ventilation, wearing garments containing phase change materials (PCM) or smart textiles that can modify the local thermal insulation or garments equipped with water cooling devices or fans could be good solutions to mitigate heat stress of electric workers [54,55,56].

#### 3.2.3. Acceptable Maximum Working Time

Electric outdoor workers are inevitably exposed to hot environments during the summer, resulting in severe heat stress. The core temperature and sweat loss are often used as indicators to assess human heat stress. In this study, the safe threshold of core temperature was set at 39 °C based on previous research [57,58,59] and the threshold of sweat loss volume was set at 5% of the body mass for grid workers based on ISO 7933 [40]. We define the time taken from starting work until the core temperature or sweat loss volume reach the threshold mentioned above as the acceptable maximum working time (AMWT) for a grid worker in a specific environment [40].

**Figure 6:**
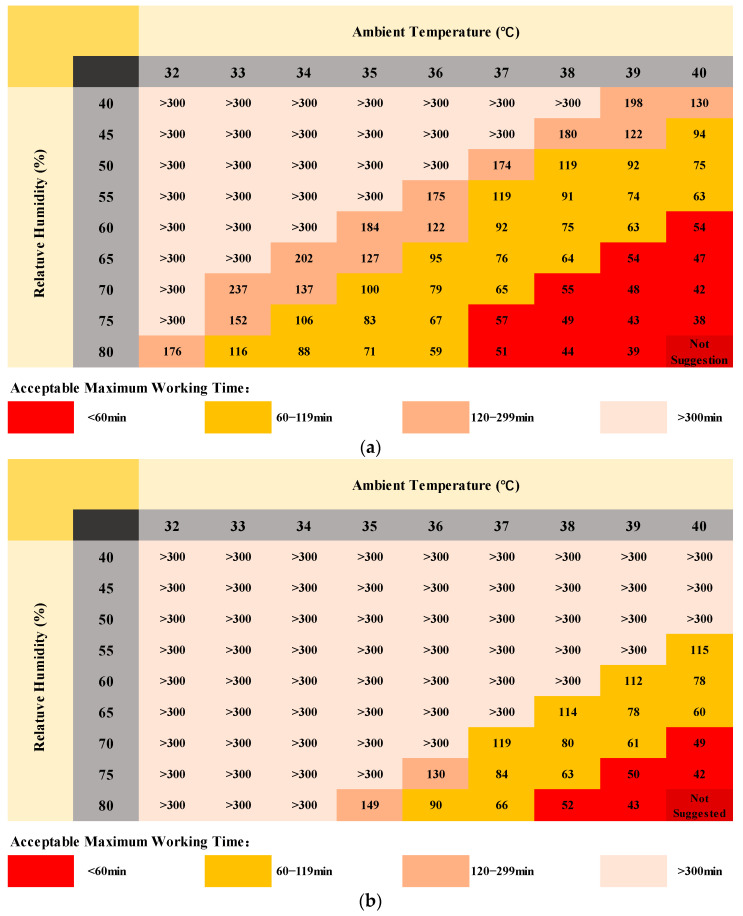

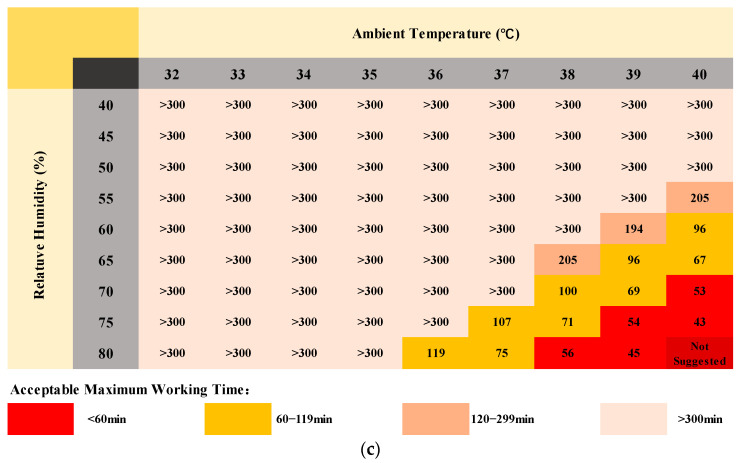
Charts of acceptable maximum working time (AMWT) for grid workers in different environments based on core temperature and sweat loss threshold: (**a**) without wind, (**b**) wind speed of 1 m/s, (**c**) wind speed of 2 m/s.

**Figure 7:**
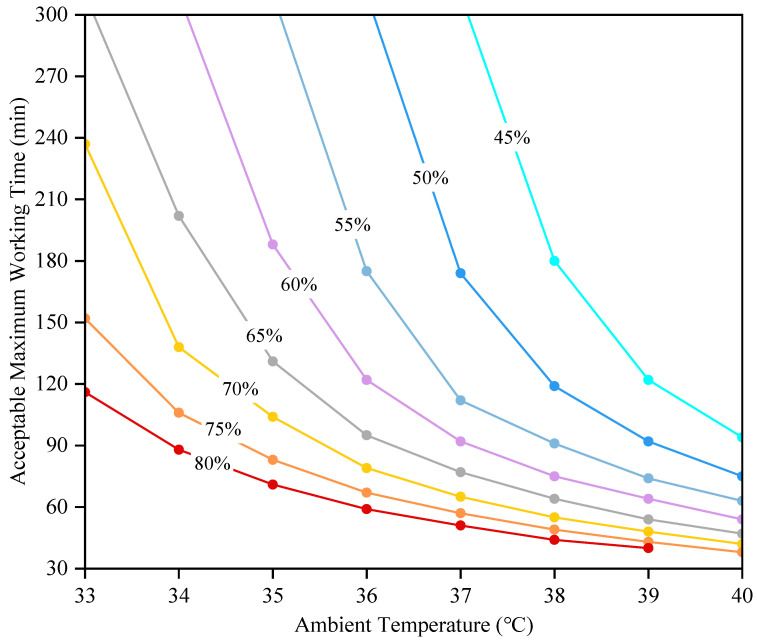
Variation of AMWT for grid workers with ambient temperature at different relative humidity (without wind).

The AMWT for grid workers working at a specific activity level (3.3 met) in different environments with wind speeds of 0.2 m/s (no wind condition), 1 m/s, and 2 m/s respectively (see Figure 6). The AMWT for grid workers decreased with the ambient temperature and humidity gradually increased. An increase in ambient temperature reduces the sensible heat loss of the human body. When the ambient temperature is close to or higher than the skin temperature, the sensible heat loss will no longer be effective and the evaporative heat loss becomes the only way to dissipate excess heat to the ambient environment, but evaporative heat loss will decrease with an increase in humidity. Therefore, under the windless conditions shown in Figure 7, the difference in AMWT for grid workers at different relative humidities became smaller as the ambient temperature increased. Taking the change process from 39 °C to 38 °C as an example, when the relative humidity was 45%, the evaporative heat loss was the main form of heat dissipation to transfer excess heat of the body to the ambient environment, and the AMWT of grid workers was also greatly increased. When the relative humidity was above 60%, the evaporative heat loss was restricted and it was difficult to effectively transfer excess heat, so the core temperature still increased and the change of AMWT was small.

In addition, comparing the AMWT at different wind speeds, the increase in wind speed effectively reduced the core temperature and thus extended the AMWT for grid workers. Overall, the change in AMWT was greater when changing from no wind to wind compared to an increase in wind speed from 1 m/s to 2 m/s. It is reasonable to assume that the compensatory effect from wind speed decreases with increasing wind speed. However, in some harsh environments with high temperature and humidity (e.g., red area in Figure 6), the cooling effect of an increased wind speed was not distinct, which was dangerous for grid workers. Another concern was the sudden change in AMWT, when AMWT changed from above 300 min to a specific value, the decline range was very large and generally higher in windy conditions than in non-windy conditions. Therefore, the AMWTs predicted by the model could have a large deviation from the actual situation if the changes in the external environment are not reflected in time. Considering the instability of parameters such as ambient temperature, humidity, and wind speed in actual outdoor environments, it is necessary to combine a human thermoregulation model with real-time weather forecast data or on-site environmental monitoring data to predict and assess the thermophysiological responses and AMWT for grid workers as accurately as possible [60].

Despite the gap with reality, the AMWT threshold charts shown in Figure 6 still have considerable significance for the prevention of heat stress for grid workers. For example, when carrying out grid inspection and maintenance, workers often operate for 4–5 h on high-altitude transmission lines. To ensure their safety and health, the threshold chart could be used to check whether the environmental conditions at the workplace can guarantee that they can work continuously for a long time so that alerts and warnings could be given in time before starting work.

### 3.3. Work Organization Advices

**Figure 8:**
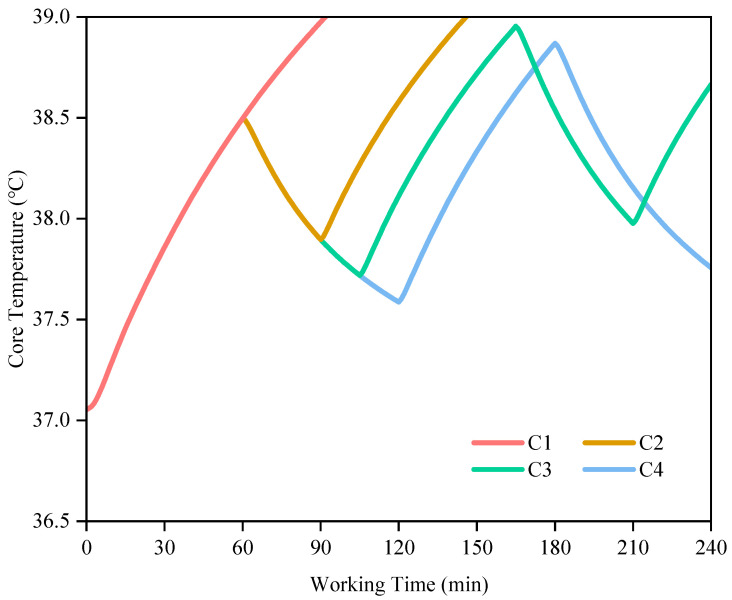
The predicted core temperature of grid workers in 240 min under different work/rest strategies (C1: a 240-min work; C2: a 30-min rest after a 60-min work; C3: a 45-min rest after a 60-min; C4: a 60-min rest after a 60-min work).

**Figure 9:**
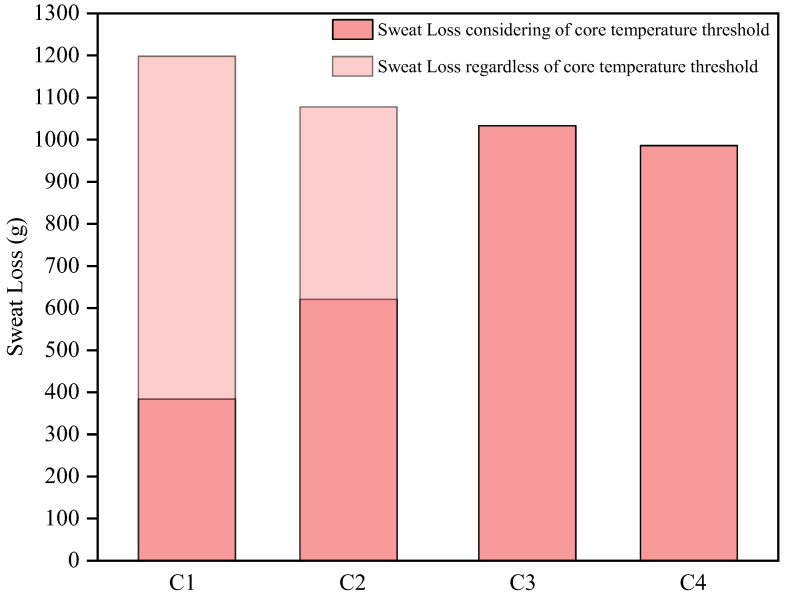
The predicted sweat loss of grid workers in 240 min under different work/rest strategies (C1: a 240-min work; C2: a 30-min rest after a 60-min work; C3: a 45-min rest after a 60-min; C4: a 60-min rest after a 60-min work).

Although there are various measures to prevent and mitigate heat stress for workers, such as ingesting ice water and wearing cooling vests, the most economically and technically feasible method for grid outdoor workers is to optimize work/rest strategies [61]. Figure 8 and Figure 9 represented the variations in core temperature and sweat loss of grid workers for four different work/rest strategies in a windless environment at a temperature of 38 °C and 55% relative humidity, respectively. Where the cases of C1, C2, C3 and C4 represented 240 min of continuous work, a 30-min rest after a 60-min work, a 45-min rest after a 60-min and a 60-min rest after a 60-min work, respectively. As the core temperature threshold for grid workers was set at 39 °C in this paper, once this threshold is reached, subsequent changes in core temperature were not be showed.

For the 240-min duration in Figure 8, the effective working time for four strategies was 91, 115, 150, and 120 min respectively. Compared to continuous work, a reasonable rest interval could considerably reduce the core temperature and thus extended the working time. In the case of C2, after a 30-min rest, the worker’s core temperature exceeded 39 °C during the second work session to the extent that the worker was unable to continue working. In this environment and activity level, a 30-min break was not sufficient for grid workers to carry out sustainable operational activities. In addition, compared to the case of C3, the core temperature of grid workers in the case of C4 decreased by 0.13 °C and 0.08 °C at the end of the first break and the end of the second work, respectively, so an additional 15-min rest did not result in a considerable decrease in the core temperature of grid workers after 120 min of work. Furthermore, the case of C3 resulted in 30 min more working time than the case of C4 over 240 min, and the work efficiency was improved based on ensuring personnel safety, so the case of C3 was considered to be a more optimal work organization strategy in this scenario. It should be noted that the core temperature of grid workers in the cases of C3 and C4 increased by 0.46 °C and 0.37 °C respectively after the second work compared with the first work. Despite a long rest, the core temperature increased by 0.67 °C and 0.53 °C after the first rest, respectively, compared with the initial time. Therefore, the 4:3 and 1:1 work-rest ratios could not restore the core temperature of grid workers to the initial level in such an environment, and it can be speculated that if such work-rest rotation is to be continued after 240 min, the core temperature of grid workers may still exceed the safety threshold of 39 °C at some time in the future.

The sweat loss of grid workers under the four work organization strategies is shown in Figure 9, where the lighter color areas in C1 and C2 represented the sweat loss of a 240-min working duration regardless of the core temperature threshold. It can be seen that the sweat loss of grid workers decreased with the prolongation of rest time. The maximum sweat loss for the four strategies was 1198 mL, 1078 mL, 1033 mL, and 986 mL respectively, a reduction of 10.0%, 13.8%, and 17.7% respectively compared to the case of C1 which was not limited by the core temperature threshold. The effective working time of the C2 case and the C4 case was 115 min and 120 min, but the difference in sweat loss was indeed 365 mL. This means that in a windless environment with Ta = 38 °C and RH = 55%, even during rest, the human body sweat profusely. Comparing the cases of C2, C3, and C4, the rest periods were 60 min, 90 min, and 120 min respectively, and the average amount of sweat was reduced by 1.53 mL per minute of rest.

## 4. Conclusions

This study measured the clothing thermal insulation of grid worker ensembles by a thermal manikin and a multi-segment bioheat model was employed to assess the thermophysiological responses of grid workers at a specific activity level in different hot environments. The main findings are presented as follows.

(a) The total insulation of power grid ensembles is 1.046 clo. The local total insulation of the torso is higher than that of the limb parts. Parts of the ensembles with high thermal insulation or skin temperature need to be considered for design improvement.
(b) The AMWT of grid workers shows obvious differences in different hot environments but approaches gradually as the ambient temperature and relative humidity increase. Wind speed can considerably extend AMWT for grid outdoor workers, but not in high temperature and humidity environments. The threshold charts of AMWT are a good evaluation tool to check whether the grid workers can work continuously for a long time in a specific environment so that alerts and warnings could be given in time.
(c) The predicted results for a specific scenario show that the core temperature of grid workers varies considerably when working with different work organization strategies. The work-rest ratio of 4:3 is optimal in the scenario, but neither it nor a 1:1 work-rest ratio makes the worker’s core temperature return to its initial level. The difference in sweat loss of grid workers is relatively small, but decreases under a longer rest interval, with an average reduction of 1.53 mL per additional minute of rest.

## Data Availability

Not applicable.
